# The rise in the contribution of denitrification is the primary reason for the increase of N_2_O emissions in the Anthropocene

**DOI:** 10.1371/journal.pone.0331712

**Published:** 2025-10-01

**Authors:** Huining Tang, Ruiyang Xia

**Affiliations:** Nanjing University of Information Science & Technology, Nanjing, China; Tennessee State University, UNITED STATES OF AMERICA

## Abstract

Nitrous oxide (N_2_O) is the fastest growing category of greenhouse gases in the recent decades. This study systematically summarized the causes of increased N_2_O emissions and proposed targeted mitigation strategies based on isotopic analysis. The increase in annual N_2_O emissions is mainly derived from microbial processes in agricultural soils and eutrophic waters, where denitrifiers are responsible for the anthropogenic emissions. In natural soil and water, minor N_2_O emissions are accompanied by a significant contribution of nitrification. Since the industrialization and urbanization, the extensive utilization and leaching of fertilizers, and the sewage discharge, have significantly facilitated the denitrification activity. For example, the contribution of denitrification to N_2_O emissions in the grassland and forest soils increased from approximately 50% to 80% after fertilization, similar phenomena have also been observed in eutrophic waters. Human disturbances enhanced N_2_O emissions by increasing the contribution of denitrification to N_2_O production. Therefore, the targeted mitigation strategies lie in the effective control of fertilizer and organic pollutant, the improvement of aeration, as well as the microbial control in fertilized soils and eutrophic waters. This study is of great significance in improving our understanding of N_2_O emission increase and provides a foundation for the development of effective mitigation strategies.

## 1. Introduction

As a powerful and long-lived (116 ± 9 years) greenhouse gas, nitrous oxide (N_2_O) is 298 times the global warming potential of CO_2_ on a 100-year timescale and it also contributes to the depletion of the stratospheric ozone layer [1]. Moreover, N_2_O is the fastest growing category of greenhouse gases in the past five years, with emissions increasing at a rate that even exceeds some of the highest projected emission scenarios [2,3]. Over the past three decades, the increase in N_2_O emissions is primarily attributable to anthropogenic sources, the proportion of which has increased from 36.1% to 42.9% (IPCC). In order to effectively predict and mitigate N_2_O emissions, it is essential to have a comprehensive understanding of the underlying processes associated with anthropogenic N_2_O sources [4].

The major anthropogenic N_2_O sources include direct emissions from agriculture (2.6 TgN yr^-1^), indirect emissions from inland and coastal waters (0.4 TgN yr^-1^), as well as fossil fuel (0.9 TgN yr^-1^) and biofuel burning (0.7 TgN yr^-1^) (IPCC AR6). Although N_2_O emissions from combustion accounted for more than 20% of the contribution, their growth has been well controlled [5]. Therefore, the direct emissions from soils and indirect emissions from waters seem to be the most significant increase responsible to anthropogenic N_2_O emissions. In soils and waters, the excessive use and inappropriate timing of N application (e.g., chemical fertilizers and manure) can lead to N_2_O emission hotspots [6]. N_2_O is mainly produced by microbial transformation of reactive nitrogen [7]. Two primary processes, i.e., nitrification and denitrification, dominate the production, reduction and emissions of N_2_O [7]. However, the quantitative identification of anthropogenic N_2_O sources and human impacts remain challenging. It severely impairs the accurate prediction of anthropogenic N_2_O emissions and constrains the development of future mitigation strategies.

The clear separation of microbial N_2_O sources is essential for the meaningful mitigation in its emissions. Stable isotope techniques have been widely used for N_2_O source partitioning in recent decades, which offer insight into N_2_O production and reduction mechanisms by determining the stable N and O isotope ratios (δ^15^N-N_2_O and δ^18^O-N_2_O) and site preference (δ^15^N^SP^, defined as δ^15^N^SP^ = δ^15^N^α^ - δ^15^N^β^) within the linear molecule (N^β^-N^α^-O) [8]. δ^15^N^SP^ value is controlled by N_2_O production processes and remains constant despite variations in the isotopic compositions of substrates (such as NO_3_^-^ and NH_4_^+^), whereas δ^15^N-N_2_O and δ^18^O-N_2_O are subject to variation [8]. The SP values of N_2_O derived from nitrification and denitrification have been determined and exhibit relatively stable ranges, i.e., from 30‰ to 36‰ for nitrification, and from −11‰ to 0‰ for denitrification, respectively. However, the isotopic signatures of N_2_O are influenced by atmosphere-water exchange [9] and reduction, which should be addressed to obtain the original isotopic values from soils and waters. Keeling plot [8,10,11] and δ^15^N^SP^/δ^18^O map approach [12] are necessary to mitigate the bias in order to subsequently determine the microbial sources of N_2_O using the mixing equation [12].

In this study, we summarize the anthropogenic N_2_O emissions and highlight the vital role of the direct and indirect emissions from soils and waters. Notably, source partitioning model based on isotopic data was established from the bottom up to quantitatively assess the N_2_O microbial sources and reveal the human impacts on N_2_O emissions. We further compared the related environmental factors between natural ecosystems and human impacted ones in order to obtain more targeted strategies for N_2_O emission mitigation. This study is vital for improving our understanding of N_2_O emissions under human activities and was in favor of the developing targeted emission mitigation strategies.

## 2. Materials and methods

### 2.1. Data collection

A literature search was conducted using bibliographic databases (e.g., Web of Science, Google Scholar, etc.) for previous studies containing N_2_O emissions and isotopic signatures that were conducted in terrestrial and aquatic systems (S1 Table, from 2000 to 2022) [13–23]. Isotopic signatures for N_2_O, i.e., δ^15^N-N_2_O, δ^18^O-N_2_O and δ^15^N^SP^-N_2_O were collected for the source partitioning of N_2_O. If the published data was not tabulated, we used the software ‘g3data’ (http://www.frantz.fi/software/g3data.php) to extract data from figures. In total, our efforts identified 316 lines data with the concentrations of N_2_O and related isotopic data.

### 2.2. Keeling plot in soils and waters

Due to the influence of atmosphere-water exchange [9], the measured isotopic signatures of N_2_O does not directly reflect the microbial sources of N_2_O. Therefore, Keeling plot was applied to obtain the isotope source signatures of N_2_O and avoid the bias [8,10,24]. The Keeling plot approach is based on the conservation of mass. It assumes that the measured isotopic signature is a mixture of background values (i.e., atmospheric N_2_O) and an addition by a source (Equations 1–3). The measured N_2_O isotopic values for each sampling point are plotted with the inverse of the observed N_2_O concentrations, and the isotopic composition of microbially produced N_2_O is represented by the y-intercept value [25]. The relevant equations are expressed as follows:


$$\left[N_{\text{2}}O_{observed}\right]=\left[N_{\text{2}}O_{background}\right]+\left[N_{\text{2}}O_{produced}\right]$$ (1)

$$\delta_{observed}*\left[N_{\text{2}}O_{observed}\right]=\delta_{background}*\left[N_{\text{2}}O_{background}\right]+\delta_{produced}*\left[N_{\text{2}}O_{produced}\right]$$ (2)

$$\delta_{observed}=\frac{\text{1}}{{\left[N_{\text{2}}O\right]}_{observed}}*\left(\delta_{background}-\delta_{produced}\right)*\left[N_{\text{2}}O_{background}\right]+\delta_{produced}$$ (3)

where [N_2_O] represents nitrous oxide concentration (nM) and δ is the isotopic composition (either δ^15^N^bulk^-N_2_O, δ^18^O-N_2_O, or δ^15^N^SP^), and the subscripts indicate whether the observed signal is from background or produced N_2_O.

### 2.3. δ^15^N^SP^/δ^18^O map approach

N_2_O reduction in ecosystems could also alter the original isotopic signatures of N_2_O. Theoretically, when the N_2_O molecule is reduced to N_2_ gas, N-O bonds with the light isotopes of nitrogen and oxygen are preferentially cleaved over bonds containing either ^15^N or ^18^O, leaving the remaining N_2_O enriched in these heavy isotopes. The direct use of the reduced isotope signatures will result in a bias towards the source partitioning of N_2_O. It has been demonstrated that the ratio between isotope effects for δ^15^N^SP^ and δ^18^O during N_2_O reduction remains relatively stable. Consequently, the δ^15^N^SP^/δ^18^O map can be used to quantify the degree of N_2_O reduction and calculate the original isotope source signatures [12,24,26]. Using the Rayleigh equation, the fraction of N_2_O reduction was calculated by the reduction line and mixing line [12]. The reduction line was defined based on the values from the literature, with a mean slope of 0.38 [12,27]. Additionally, a mixing line is drawn between the mean values for both δ^15^N^sp^ and δ^18^O of the respective process (S2 Table). The intersection between the reduction line and the mixing line was used for the subsequent source partitioning of N_2_O. As outlined in previous studies [12,27], Further details concerning the utilisation of the δ^15^N^SP^/δ^18^O map method for the identification of microbial sources of N_2_O and the quantification of N_2_O reduction degree are available.

### 2.4. Microbial source partitioning of N_2_O

The distance between two endmember values at the intersection point represents the contribution of each microbial process. According to δ^15^N^SP^ signatures from nitrification (NFT) and denitrification (DFT), the relative contribution ratios of different sources can be calculated following Equations 4 and 5:


$$\delta^{15}{N^{SP}}_{,original}\;=\;\delta^{15}{N^{SP}}_{,NFT}\;\times \;f_{NFT}\;+\;\delta^{15}{N^{SP}}_{,DFT}\;\times \;f_{DFT}$$ (4)

$$f_{NFT}\;+\;f_{DFT}\;=1$$ (5)

where δ^15^N^SP^_, original_ denotes the δ^15^N^sp^ of the mixed N_2_O before reduction, δ^15^N^SP^_, NFT_ is the initial δ^15^N^sp^ for nitrification, δ^15^N^SP^_, DFT_ is the initial δ^15^N^sp^ for bacterial denitrification, *f*_NFT_ is the fraction of nitrification-derived N_2_O to microbial-derived N_2_O, *f*_DFT_ is the fraction of denitrification-derived N_2_O to microbial-derived N_2_O.

### 2.5. Statistical analysis

The study conducted a one-way analysis of variance (ANOVA) to examine statistically significant differences among the various sampling sites. Pearson correlation analysis was performed to evaluate the relationships between N_2_O concentrations and isotopic signatures. The t-test was used to compare the linear regression slopes. The one-way ANOVA, Pearson’s correlation analysis, and t-test were performed using the SPSS 18.0 statistical software package.

## 3. Results and discussion

### 3.1. Significant increase of anthropogenic N_2_O emissions

**Fig 1:**
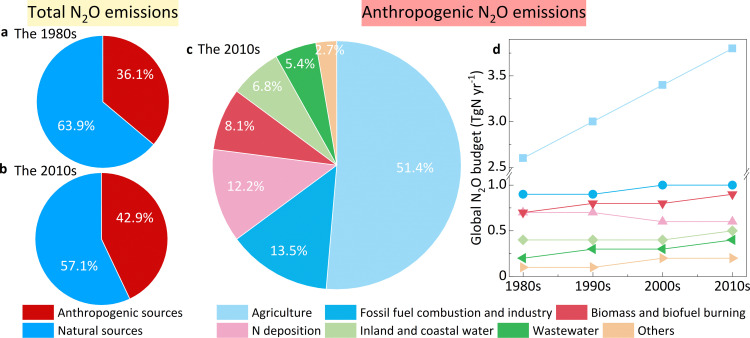
Natural and anthropogenic N_2_O emissions from the 1980s to the 2010s. (a) Proportion of natural and anthropogenic sources of N_2_O in the 1980s. (b) Proportion of natural and anthropogenic sources of N_2_O in the 2010s. (c) Proportion of different anthropogenic sources in the 2010s. (d) Global budget of anthropogenic N_2_O emissions from the 1980s to the 2010s. Data sources: IPCC.

Using bottom-up (inventory, flux measurements, process-based modelling) and top-down (atmospheric inversion) approaches, it has been determined that global annual N_2_O emissions have increased from 15.5 TgN yr ⁻ ¹ (in the 1980s) to 17.0 TgN yr ⁻ ¹ (in the 2010s) in the past three decades (IPCC 6AR). This rise (1.5 TgN yr^-1^) is primarily attributed to the anthropogenic sources, the proportion of which has increased from 36.1% to 42.9% (Fig 1a, b). The primary anthropogenic contributors include agriculture (51.4%), fossil fuel combustion and industry (13.5%), N deposition (12.2%), Biomass and biofuel burning (8.1%), inland and coastal waters (6.8%), and wastewaters (5.4%) (Fig 1c).

Among these anthropogenic sources, agricultural N_2_O emissions was the primary contributor (51.4%) in the decade 2010s (Fig 1c). Moreover, it increased steadily from the 1980s (2.6 TgN yr ⁻ ¹) to the 2010s (3.8 TgN yr ⁻ ¹) and exhibited the highest growth rate (Fig 1d). Similarly, N_2_O from inland and coastal waters also continuously increased. As an indirect emission from agricultural activities, its emission level exhibited a concomitant increase with agricultural emissions. Additionally, global N deposition could also stimulate N_2_O emissions from soils and waters by microbial processes. Based on terrestrial biosphere models, the continued increase in wastewater N_2_O emissions from domestic and industrial sources have shown a gradual increase, consequently raising their share of anthropogenic emissions. Notably, although N_2_O emissions from fossil fuel (13.5%) and biofuel (8.1%) burning accounted for more than 20% of the contribution, they were found to be well controlled and gradually decreased owing to the optimization of energy structures and the advancement of technologies (Fig 1d). Therefore, soils and waters are responsible for the most significant increase in anthropogenic N_2_O emissions, where microbial processes dominate N_2_O productions and emissions.

### 3.2. Human activities significantly altered microbial sources of N_2_O

Nitrification and denitrification are two key processes controlling N_2_O production and emissions in soils and waters [5]. δ^15^N^SP^ is independent of substrate, the use of which can differentiate N_2_O pathways between nitrification and denitrification [28]. Higher δ^15^N^SP^ values indicate a greater contribution of nitrification, while lower values specify denitrification [29]. Therefore, this study has conducted a correlation analysis between N_2_O concentrations and δ^15^N^SP^ values to reveal the impact of human activities on microbial N_2_O sources.

**Fig 2:**
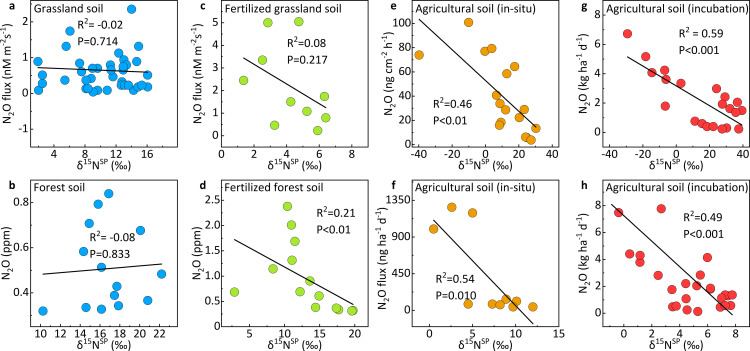
Correlations between N_2_O emissions (e.g., concentration or flux) and δ^15^N^SP^ values. Grassland soil (a, n = 40), forest soil (b, n = 14), fertilized grassland soil (c, n = 10), fertilized forest soil (d, n = 14), in-situ agricultural soil (e, n = 17; f, n = 10), and lab-cultured agricultural soil (g, n = 21; h, n = 28).

**Fig 3:**
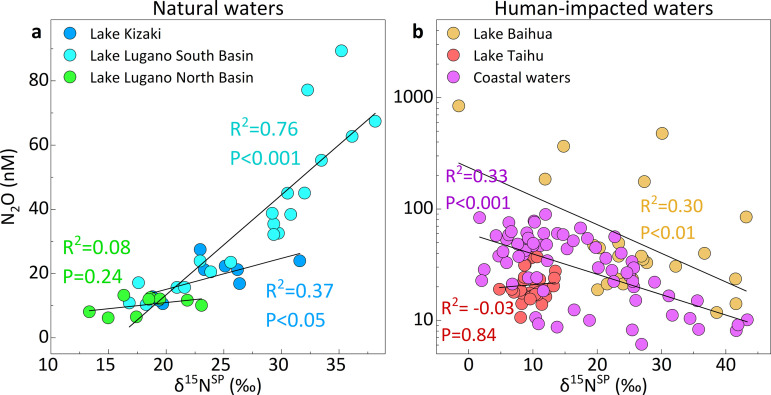
Correlations between N_2_O concentrations and SP values. Natural waters (a, n = 39) and human-affected waters (b, n = 123).

In natural soils with minimal human interference [17,18], e.g., grassland and forest soils, there was no significant correlation between N_2_O emissions and δ^15^N^SP^ values (p > 0.05) (Fig 2a, b). This indicates the diverse microbial sources of N_2_O in natural soils, including both nitrification and denitrification. However, a notable increase in N_2_O emissions has been observed after fertilizer addition, accompanied by negative correlations between N_2_O emissions and δ^15^N^SP^ values (Fig 2c, d). δ^15^N^SP^ values decreased from 10.3‰ to 4.3‰ in grassland soils and from 16.8‰ to 13.2‰ in forest soils. As lower δ^15^N^SP^ values indicate a greater contribution of denitrification, anthropogenic activities likely stimulate N_2_O emissions by promoting denitrification activities. Moreover, continuous agricultural activity over the years has led to a significant negative correlation between N_2_O emissions and δ^15^N^SP^ values in situ (Fig 2e, f, p < 0.01), with much lower δ^15^N^SP^ values compared to natural soils [19,20]. When agricultural soils were cultured in the laboratory [21,22], the significance of the negative correlation further increased (Fig 2g, h p < 0.001). A similar phenomenon has been observed in waters. In natural lakes and rivers, N₂O concentrations were positively correlated with δ^15^N^SP^ values (Fig 3a) [13,14,30], indicating nitrification is the primary process responsible for N₂O production. Conversely, in eutrophic lakes and urban rivers, N₂O concentrations exhibited a significant negative correlation with δ^15^N^SP^ values (Fig 3b), suggesting that denitrification predominates in these environments [8,15,16,31]. Moreover, N_2_O concentrations significantly increased in anthropogenically disturbed waters, revealing the key contribution of denitrification to anthropogenic N_2_O emissions. Overall, the N_2_O emissions and the significance of negative correlation continually increased due to the anthropogenic disturbances, along with the rise of the contribution of denitrification.

In soils and waters, microbial N_2_O sources are intricately influenced by environmental factors, such as organic carbon content, substrate concentrations (NH_4_^+^ and NO_3_^−^) and dissolved oxygen (DO). Human interference has disrupted the original ecological balance, promoting the excessive N_2_O emissions with the shift from nitrification to denitrification in microbial N₂O pathways. In human-impacted soils, the introduction of nitrate and organic matter provides substrates for denitrification [32], regular agricultural irrigation also increases the water filled pore space (WFPS) of soils and creates anaerobic environments [21], leading to higher denitrification activities and lower the δ^15^N^SP^ values. Given that O_2_ concentration is usually inversely proportional to the levels of water filled pore space (WFPS) and depth, δ^15^N^SP^ values of N₂O in agricultural soils likely decline as WFPS and soil depth increase. In natural waters, high oxygen level ensures nitrification. Several studies have found that δ^15^N^SP^ values increase with depth in natural water bodies [13,14,30]. This may be attributed to the persisting aerobic conditions, higher NH_4_^+^ availability and the recovery of AOB from photoinhibition at greater depths [13,30]. Due to the irreversible damage to ammonia monooxygenase, the extent of photoinhibition and the recovery time for AOB depend on light wavelength, intensity and photon quantity, all of which are affected by water depth [33]. In eutrophic waters, the substantial negative correlation between δ^15^N^SP^ values and depth can be attributed to the increased anthropogenic input of organic matter and subsequent anaerobic layer in eutrophic waters [15,34]. As oxygen concentration decreases with depth, denitrification increases, causing the bottom of the water column to exhibit minimal values of δ^15^N^SP^ values [15]. Notably, N₂O might be further reduced to N₂ under more anoxic conditions, leading to a significant increase in δ^15^N^SP^ values with depth [8,13,16]. All of the above results indicate that the extent of human disturbances can influence the contribution of the denitrification process, and that the increase in denitrification is the main reason for the rise in microbial sources producing N_2_O.

### 3.3. Quantification of microbial sources of N_2_O

**Fig 4:**
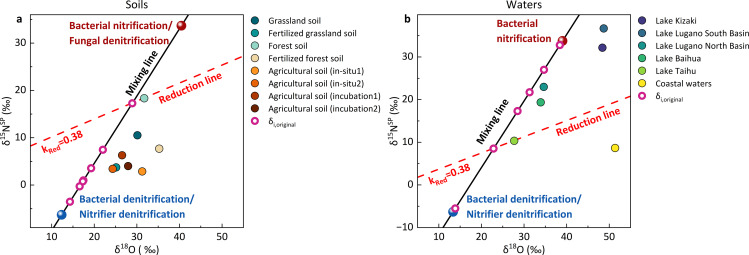
Schematic diagram of the δ^15^N^SP^/δ^18^O map approach in soils (a) and waters (b). The mixing line was drawn between the average values of the major two microbial process, nitrification and denitrification. Another reduction line with a mean slope of 0.38 was defined based on the values from the literature [27]. The solid circles represent the N_2_O isotope values in each sampling site after reduction, and the purple circles represent the original N_2_O isotope values before reduction. The mean and standard deviations for each microbial process and isotopic values of precursor are summarized in S2 Table.

**Fig 5:**
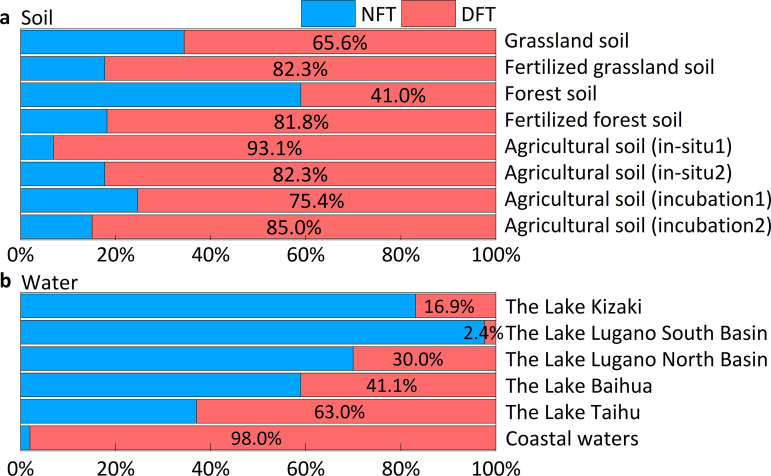
Quantification of different microbial sources in waters (a), and soils (b). NFT and DFT indicate nitrification and denitrification.

The atmosphere-water exchange and N_2_O reduction in ecosystems could alter the original isotopic signatures of N_2_O (δ_i, original_), thus we applied Keeling plot (S1 and S2 Figs) and δ^15^N^SP^/δ^18^O map approach (Fig 4) to eliminate the bias derived from atmosphere-water exchange and N_2_O reduction [24,26]. After obtaining the corrected δ^15^N^SP^ values of N_2_O (δ_i, original_), the microbial contribution of N_2_O was quantified based on the conservative δ^15^N^SP^ signatures from nitrification and denitrification (Fig 5).

In the grassland and forest soils, the contribution of denitrification to N_2_O emissions increased from approximately 50% to 80% after fertilization (Fig 5a). Moreover, the dominant role of denitrification was more stable in agricultural soils, either in-situ or in laboratory, ranging from 75.4% to 93.1% (Fig 5a). The variation is related to soil type, fertilization type and quantity, or experimental conditions. In natural waters, the contributions of nitrification were significantly larger than those of denitrification, with denitrification accounting for only 16.9% in Lake Kizaki, 2.4% in Lake Lugano South Basin, and 30.0% in Lake Lugano North Basin, respectively (Fig 5b). As anthropogenic impacts rise, denitrification gradually became the dominant process in eutrophic waters because of the abundant organic electron donors and microoxic environment. The contribution of denitrification accounts for 41.1% in Lake Baihua, 63.0% in Lake Taihu, and 98.0% in the coastal waters (Fig 5b), significantly higher than that in natural waters (p < 0.01). Overall, the contribution of denitrification significantly increased in soils and waters with long-term anthropogenic impacts (p < 0.05). These quantitative results from soils and waters provide solid evidence that human disturbances can enhance N_2_O emissions by increasing the contribution of denitrification to N_2_O production. The increase in annual N_2_O emissions is primarily attributed to the anthropogenic sources, which are mainly derived from denitrification.

### 3.4. Targeted mitigation strategies

**Fig 6:**
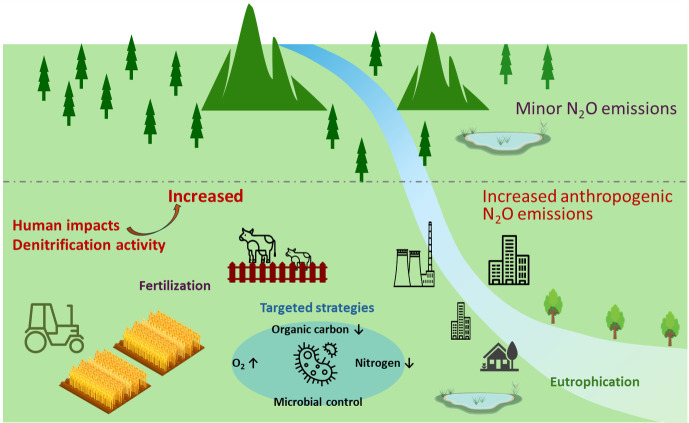
The increased anthropogenic N_2_O emissions and targeted strategies in soils and waters. Reprinted from [35] under a CC BY license, with permission from Oxford University Press, original copyright 2024.”.

Anthropogenic sources from soils and waters are responsible to the significant increase of N_2_O emissions in recent decades, which is primarily driven from denitrification. Therefore, the effective control of biogenic material (e.g., C and N) and the regulation of redox conditions (i.e., oxygen) are in favor of emission mitigation of N_2_O by affecting microbial activity (Fig 6). There are numerous mitigation options in the soils and waters. Firstly, scientific fertilization in agriculture, including the increasing efficiency of nitrogen use, split applications, and the optimization of fertilization time. The adoption of best management practices (BMPs) can potentially increase crop nitrogen recovery [36], which can be achieved through the precise estimation of crop needs and the application of slow- and controlled-release fertilizer forms [36]. Studies indicate that crop-rotation management and the use of catch- or cover-crops can significantly reduce the need for chemically derived nitrogen fertilizer [36]. Furthermore, the placement of fertilizer nitrogen into the soil near the zone of active root uptake, the use of appropriate nitrogen rates, and synchronizing the timing of fertilizer nitrogen application with plant nitrogen demand can also improve nitrogen fertilizer use efficiency, thereby mitigating N_2_O emissions [36]. Secondly, it has been suggested that the application of organic amendments and fermented organic manure reduce N_2_O emissions from agricultural land [1]. However, organic matter could serves as a source energy for denitrification and accelerates the formation of anaerobic conditions, inversely increases N_2_O emissions [1,37]. Our results indicate that the increased anthropogenic N_2_O sources is primarily driven from denitrification, and the organic fertilizer might increase N_2_O emissions by promoting the activity of denitrification. Thus, it is crucial to carefully manage and, when necessary, reduce anthropogenic inputs of organic matter. Additionally, high levels of WFPS and deeper soil layers are conducive to the formation of anaerobic conditions, which promote denitrification. It has been proposed that constructing a well-developed drainage system to prevent over-wetting of farmland, along with deep loosening of the soil to improve aeration, can enhance the O_2_ content in the soil, thereby reducing N_2_O emissions resulting from denitrification [1,38–40]. In eutrophic waters, in addition to the control of agricultural non-point source pollution mentioned above, it is also needed to control urban point source pollution. Reducing the organic pollutants input, as well as the algal bloom caused by the N/P input, could avoid the formation of anaerobic state and the emission of N_2_O mainly by denitrification. Moreover, microbial control is also the key to mitigating N_2_O emissions. Hiis et al. demonstrated that applying non-denitrifying N_2_O-respiring bacteria is a feasible approach to mitigate denitrification-derived N_2_O emissions in soils [41]. For example, *Cloacibacterium sp. CB-01* is considered to be a promising strain, of which the genome contains nosZII but lacks any genes coding for the reduction of NO_3_^−^ and NO_2_^−^ [41]. Several studies also proposed that nitrification inhibitors could reduce the production of N_2_O from ammonia oxidation [42,43]. The inhibition of ammonia oxidation also benefits crop nitrogen uptake [44]. Based on our results, denitrification is the main microbial process responsible for the increased N_2_O emissions in agricultural fields. Nitrification inhibitors might play a greater role in the systems with less human impact or better ventilation conditions.

## 4. Conclusion

This study demonstrated that the isotopic signatures of N_2_O are valuable indicators to quantify N_2_O microbial sources, which supports the development of effective mitigation strategies. Our findings emphasize the critical role of anthropogenically-driven denitrification in promoting microbial N₂O emissions. The observed increase in global annual N_2_O emissions is primarily attributed to the enhanced microbial processes in agricultural soils and eutrophic waters. Furthermore, the microbial sources exhibited a continuous growth trend, thereby the mitigation of which is a priority to reduce atmospheric N_2_O concentration. The quantified results based on isotopes indicate that denitrification gradually became the dominant process in fertilized soils and eutrophic waters as a consequence of rising anthropogenic impacts. Therefore, the effective control of fertilizer and organic pollutants, the improvement of aeration, and microbial control are in favor of the N_2_O emission mitigation. Although the typical medias (i.e., soils and waters) with varying degrees of human influence in this article does have certain representativeness, more in situ isotope measurements are necessary to constrain the highly variable environments and derive more reliable conclusions.

## Supporting information

S1 Table Data source for isotopic signatures of N_2_O. (DOCX)

S1 Fig Keeling plot analysis of N_2_O sources. Grassland soil (a), fertilized grassland soil (b), in-situ agricultural soil (c, g), lab-cultured agricultural soil (d, h), forest soil (e), and fertilized forest soil (f).
(TIF)

S2 Fig Keeling plot analysis of N_2_O sources. Lake Kizaki (a), Lake Lugano South Basin (b), Lake Lugano North Basin (c), Lake Baihua (d), Lake Taihu (e), and coastal waters (f).
(TIF)

S2 Table The mean and standard deviations for each microbial process. (DOCX)
