# Supplementary material for: The rise in the contribution of denitrification is the primary reason for the increase of N2O emissions in the Anthropocene
Source: PLoS One. 2025 Oct 1;20(10):e0331712. doi: 10.1371/journal.pone.0331712 (PMC12488026; doi:10.1371/journal.pone.0331712)
Supplement: S1 Table — (DOCX) [file pone.0331712.s001.docx]

S1 Table. Data source for isotopic signatures of N_2_O.

| **Related data** | **Sites** |
| --- | --- |
| Grassland soil | Switzerland [1] |
| Fertilized grassland soil |  |
| Forest soil | Republic of Panama [2] |
| Fertilized forest soil |  |
| Agricultural soil (In-situ1) | Venezuela [3] |
| Agricultural soil (In-situ2) | Japan [4] |
| Agricultural soil (incubation1) | UK [5] |
| Agricultural soil (incubation2) | UK [6] |
| Natural waters (Lake Kizaki) | Japan [7] |
| Natural waters (Lake Lugano South Basin) | Switzerland [8] |
| Natural waters (Lake Lugano North Basin) | Switzerland [8] |
| Human-impacted waters (Lake Baihua) | China [9] |
| Human-impacted waters (Lake Taihu) | China [10] |
| Human-impacted waters (Coastal waters) | Peru coast [11] |

**Reference:**

1. Wolf B, Merbold L, Decock C, Tuzson B, Harris E, Six J, et al. First on-line isotopic characterization of N_2_O above intensively managed grassland. Biogeosciences. 2015;12: 2517–2531. doi:10.5194/bg-12-2517-2015

2. Koehler B, Corre MD, Steger K, Well R, Zehe E, Sueta JP, et al. An in-depth look into a tropical lowland forest soil: nitrogen-addition effects on the contents of N2O, CO2 and CH4 and N2O isotopic signatures down to 2-m depth. Biogeochemistry. 2012;111: 695–713. doi:10.1007/s10533-012-9711-6

3. Park S, Pérez T, Boering KA, Trumbore SE, Gil J, Marquina S, et al. Can N2O stable isotopes and isotopomers be useful tools to characterize sources and microbial pathways of N2O production and consumption in tropical soils? Global Biogeochemical Cycles. 2011;25. doi:10.1029/2009GB003615

4. Maeda K, Toyoda S, Shimojima R, Osada T, Hanajima D, Morioka R, et al. Source of nitrous oxide emissions during the cow manure composting process as revealed by isotopomer analysis of and amoA abundance in betaproteobacterial ammonia-oxidizing bacteria. Appl Environ Microbiol. 2010;76: 1555–1562. doi:10.1128/AEM.01394-09

5. Meijide A, Cardenas LM, Bol R, Bergstermann A, Goulding K, Well R, et al. Dual isotope and isotopomer measurements for the understanding of N2O production and consumption during denitrification in an arable soil. European Journal of Soil Science. 2010;61: 364–374. doi:10.1111/j.1365-2389.2010.01233.x

6. Bergstermann A, Cárdenas L, Bol R, Gilliam L, Goulding K, Meijide A, et al. Effect of antecedent soil moisture conditions on emissions and isotopologue distribution of N2O during denitrification. Soil Biology and Biochemistry. 2011;43: 240–250. doi:10.1016/j.soilbio.2010.10.003

7. Sasaki Y, Koba K, Yamamoto M, Makabe A, Ueno Y, Nakagawa M, et al. Biogeochemistry of nitrous oxide in Lake Kizaki, Japan, elucidated by nitrous oxide isotopomer analysis. Journal of Geophysical Research: Biogeosciences. 2011;116. doi:10.1029/2010JG001589

8. Wenk CB, Frame CH, Koba K, Casciotti KL, Veronesi M, Niemann H, et al. Differential N2O dynamics in two oxygen-deficient lake basins revealed by stable isotope and isotopomer distributions. Limnology and Oceanography. 2016;61: 1735–1749. doi:10.1002/lno.10329

9. Yue F-J, Li S-L, Liu C-Q, Mostofa KMG, Yoshida N, Toyoda S, et al. Spatial variation of nitrogen cycling in a subtropical stratified impoundment in southwest China, elucidated by nitrous oxide isotopomer and nitrate isotopes. Inland Waters. 2018;8: 186–195. doi:10.1080/20442041.2018.1457847

10. Liang X, Wang B, Gao D, Han P, Zheng Y, Yin G, et al. Nitrification Regulates the Spatiotemporal Variability of N2O Emissions in a Eutrophic Lake. Environ Sci Technol. 2022;56: 17430–17442. doi:10.1021/acs.est.2c03992

11. Bourbonnais A, Letscher RT, Bange HW, Échevin V, Larkum J, Mohn J, et al. N2O production and consumption from stable isotopic and concentration data in the Peruvian coastal upwelling system. Global Biogeochemical Cycles. 2017;31: 678–698. doi:10.1002/2016GB005567
