# Supplementary material for: The rise in the contribution of denitrification is the primary reason for the increase of N2O emissions in the Anthropocene
Source: PLoS One. 2025 Oct 1;20(10):e0331712. doi: 10.1371/journal.pone.0331712 (PMC12488026; doi:10.1371/journal.pone.0331712)
Supplement: S2 Table — (DOCX) [file pone.0331712.s004.docx]

S2 Table. The mean and standard deviations for each microbial process.

| Literature values | | |
| --- | --- | --- |
| Process | ↋^18^O (‰) | δ^15^N^SP^ (‰) |
| Denitrification [12] | 19.0 ± 2.1 | -1.9 ± 4.6 |
| Nitrifier denitrification [13] | 15.7 ± 2.9 | -10.7 ± 2.9 |
| Nitrification [14] | 39.0 ± 2.9 | 33.8 ± 4.3 |
| Fungal denitrification [15] | 46.9 ± 3.8 | 33.6 ± 2.5 |

Notes: the literature values are obtained from previous research. Specifically, the corrected δ^18^O-N_2_O for denitrification, nitrifier denitrification and fungal denitrification depend on the values of δ^18^O-H_2_O in the literature.

**Reference:**

12. Lewicka-Szczebak D, Lewicki MP, Well R. N_2_O isotope approaches for source partitioning of N_2_O production and estimation of N_2_O reduction – validation with the ^15^N gas-flux method in laboratory and field studies. Biogeosciences. 2020;17: 5513–5537. doi:10.5194/bg-17-5513-2020

13. Frame CH, Casciotti KL. Biogeochemical controls and isotopic signatures of nitrous oxide production by a marine ammonia-oxidizing bacterium. Biogeosciences. 2010;7: 2695–2709. doi:10.5194/bg-7-2695-2010

14. Sutka RL, Ostrom NE, Ostrom PH, Breznak JA, Gandhi H, Pitt AJ, et al. Distinguishing Nitrous Oxide Production from Nitrification and Denitrification on the Basis of Isotopomer Abundances. Appl Environ Microbiol. 2006;72: 638–644. doi:10.1128/AEM.72.1.638-644.2006

15. Chen X, Zhang S, Liu J, Wang J, Xin Y, Sun S, et al. Tracing Microbial Production and Consumption Sources of N2O in Rivers on the Qinghai-Tibet Plateau via Isotopocule and Functional Microbe Analyses. Environ Sci Technol. 2023;57: 7196–7205. doi:10.1021/acs.est.3c00950
